# Parent-child (dis)agreement in patient-reported outcomes in juvenile idiopathic arthritis: the role of child depression and caregiver burden

**DOI:** 10.1016/j.ero.2026.100253

**Published:** 2026-09-22

**Authors:** Michelle Dey, Lisa Pedrotti, Federica Vanoni, Tatjana Welzel, Julia Dratva, Rotraud K. Saurenmann, PAVE consortium

**Affiliations:** a Department of Health, Institute of Public Health, Zurich University of Applied Sciences (ZHAW), Winterthur, Switzerland; b Institute of Pediatrics of Southern Switzerland, Ente Ospedaliero Cantonale, Bellinzona, Switzerland; c Faculty of Biomedical Sciences, Università della Svizzera Italiana, Lugano, Switzerland; d Pediatric Rheumatology, University Children’s Hospital Basel (UKBB), University of Basel, Switzerland; e Pediatric Research Center, University Children’s Hospital Basel (UKBB), University of Basel, Switzerland; f Medical Faculty, University of Basel, Basel, Switzerland; g Cantonal Hospital Winterthur, Center for Pediatrics, Switzerland

## Abstract

**Objectives:**

Patient-reported outcome measures (PROMs) are essential for capturing disease impact and supporting patient-centred care (PCC). In paediatric settings, proxy reports (proxy-PROMs [P-PROMs]) are often used alongside or instead of self-reports. This study aimed to assess the agreement between PROMs and P-PROMs, examine group-level differences, and identify factors associated with disagreement in juvenile idiopathic arthritis (JIA).

**Methods:**

Data were drawn from a Swiss multicentre cross-sectional study. Eighty-eight children with JIA aged 10 to 18 years and their parents completed self- and proxy-reported questionnaires. Agreement at the individual level was assessed using intraclass correlation coefficients for Juvenile Arthritis Multidimensional Assessment Report (JAMAR) and Patient-Reported Outcomes Measurement Information System outcomes. Group-level differences were examined using paired t tests or Wilcoxon signed-rank tests with effect sizes (Cohen’s *d* or *r*). Mean difference scores (parent minus child) for physical and psychosocial health-related quality of life (HRQOL), assessed within the JAMAR, were categorised as agreement, child-worse, or parent-worse. Multinomial regression analyses (reference category: agreement) were used to identify predictors of disagreement.

**Results:**

Agreement at the individual level was predominantly moderate. No consistent group-level differences were observed across outcomes. Higher child-reported depressive symptoms were associated with an increased likelihood of children reporting worse psychosocial and physical HRQOL, whereas higher caregiver burden was associated with a greater likelihood of parents reporting worse HRQOL.

**Conclusions:**

Child-parent agreement in JIA is moderate, underscoring the importance of integrating both perspectives in clinical assessment and personalised care. Higher levels of child depressive symptoms and caregiver burden were associated with poorer self- and proxy-reported HRQOL, respectively, and should be considered when interpreting parent-child disagreement and guiding individualised PCC.

**WHAT IS ALREADY KNOWN ON THIS TOPIC**

• Parent proxy-reports and child self-reports in paediatric rheumatology show only moderate agreement, with limited and inconsistent evidence regarding group-level differences and factors associated with disagreement.

**WHAT THIS STUDY ADDS**

• This study demonstrates that parent-child agreement in juvenile idiopathic arthritis is predominantly moderate without consistent group-level differences, and that disagreement is associated with child depressive symptoms and caregiver burden.

**HOW THIS STUDY MIGHT AFFECT RESEARCH, PRACTICE OR POLICY**

• These findings support the routine integration of both parent and child perspectives and highlight disagreement as a clinically meaningful indicator that may improve the interpretation of patient-reported outcomes and guide individualised patient-centred care.

Alt-text: Unlabelled box dummy alt text

## INTRODUCTION

Juvenile idiopathic arthritis (JIA) is a chronic, painful inflammatory disease with onset in childhood, which can affect joints, tendons, and eyes. Most patients require long-term medication to control their disease activity and may face disease flares over time. Uncontrolled disease activity may lead to functional limitations, visual loss, and joint destruction [1]. In addition to clinical and laboratory outcome parameters, patient-reported outcome measures (PROMs) are crucial for understanding the impact of the disease and its treatment on affected children and adolescents [2]. However, as self-reports are often unavailable for young patients, they are supplemented by proxy reports (proxy-PROMs [P-PROMs]), usually assessed by parents [3].

In line with the broader literature [[2], [3], [4], [5], [6], [7]], studies performed in paediatric rheumatology generally report poor to moderate, and occasionally high, correlations and agreement (most commonly assessed using intraclass correlation coefficients, ICC) between children and proxies at the individual level [[8], [9], [10], [11], [12], [13], [14], [15], [16], [17], [18], [19]]. Correlations and agreement are typically higher in observable and functional domains (eg, physical functioning and daily activities) and lower in more subjective domains (eg, emotional and psychosocial functioning) [8,14,15,19]. In contrast, evidence on group-level differences between parent and child reports (usually measured by t tests or nonparametric equivalents) remains inconsistent: some JIA studies suggest a tendency towards more negative proxy compared with self-ratings [9,20], whereas others document both over- and underestimation by proxies [[10], [11], [12],^21^], gender- [22], and/or domain-specific differences between PROMs and P-PROMs [17], or no significant differences at all [16,19,23].

In terms of predictors, more severe disease manifestations (including greater pain [9], more activity restrictions [10], higher overall disease severity [11], more pronounced functional limitations [12], and higher active joint counts [23]) have repeatedly been associated with greater disagreement between proxy/parent and child self-reports. However, other JIA studies have reported higher agreement in the context of more severe or active disease [14,16], as well as at both mild and severe levels of disease compared with moderate levels, which may be more difficult for parents to interpret [21]. In addition to clinical JIA characteristics, child depressive symptoms [11,12] and negative mood more generally [10] have been linked to increased disagreement. Evidence from the broader paediatric literature further indicates that parental emotional distress may contribute to more negative parental perceptions of children’s health-related quality of life (HRQOL) [2], thereby potentially reducing agreement. Sociodemographic variables and child characteristics such as sex/gender and age have likewise been discussed as potential predictors of agreement in reviews [4,6] and JIA studies [8,9,16,[21], [22], [23]], although findings remain inconclusive.

Overall, evidence regarding parent-child agreement in PROMs remains insufficiently understood in general and specifically for JIA. Therefore, the present study aimed to (1) assess agreement between PROMs and P-PROMs; (2) examine group-level differences; and (3) identify factors associated with disagreement in JIA. Given that JIA affects multiple domains of functioning and well-being, agreement may vary depending on whether outcomes are more observable or more subjective. Therefore, a broad range of outcomes was used to evaluate domain-specific agreement patterns and differences between PROMs and P-PROMs. In contrast, regression analyses focused on physical and psychosocial HRQOL as integrative indicators of overall well-being, enabling the identification of predictors of disagreement at a more general level.

Based on the existing literature, we hypothesised that (1) agreement at the individual level would be lower in less observable domains (eg, anxiety, depressive symptoms, psychosocial HRQOL, and pain) and higher in more observable domains (eg, physical functioning and physical HRQOL); (2) no consistent pattern of systematic group-level differences between parent and child reports would emerge; and (3) greater disagreement in psychosocial and physical HRQOL would be associated with higher clinician-rated disease activity, greater caregiver burden, and higher levels of child-reported depressive symptoms.

## METHODS

### Study procedure

This Swiss multicentre, cross-sectional survey study was conducted within the international PAVE project (Producing an Arthritis Value-Framework with Economic Evidence – Paving the Way for Rare Childhood Disease), a collaborative initiative that investigates the socioeconomic impact of JIA involving partners from Canada, Germany, Switzerland, Spain, Israel, and Belgium [24].

In Switzerland, children and adolescents with JIA and their parents were recruited from all 9 paediatric rheumatology outpatient clinics in the German- and Italian-speaking regions (see Supplementary for details). Parents of children aged 0 to 18 years with a confirmed diagnosis of JIA, according to the International League of Associations for Rheumatology criteria [25], were eligible to participate. Patients aged 10 to 18 years were additionally invited to participate if at least 1 parent or legal guardian also consented to study participation. Adolescents aged ≥14 years provided consent themselves, whereas for children aged 10 to 13 years, consent was obtained from their parents or legal guardians.

For eligible participants, clinicians at the recruiting centres entered clinical and sociodemographic data on the children/adolescents and their parents into a Research Electronic Data Capture database [26,27] hosted at the Zurich University of Applied Sciences. Subsequently, personalised survey links were automatically sent to parents and, where applicable, to participating children and adolescents with JIA. Participants completed the online survey. Study participation ended upon survey completion. Automated email reminders were sent after 2 and 4 weeks, if necessary. In the event of continued nonresponse or incomplete questionnaires, clinicians at the participating centres would follow up directly with participants. The study was approved by the lead ethics committee (Cantonal Ethics Committee Zurich) and by the respective ethics committees responsible for all participating study sites (Business Adminstration System for Ethics Committees No. 2023-01069).

### Variables of interest

#### Clinical and sociodemographic characteristics

Clinicians provided sociodemographic information about the child/adolescent, including age and gender identity, as well as clinical data, including JIA subtype and the physician’s global assessment of disease activity, assessed with a visual analogue scale (VAS) ranging from 0 ‘inactive’ to 10 ‘highly active’.

#### Patient- and parent-reported outcomes

PROMs and P-PROMs were assessed using the Juvenile Arthritis Multidimensional Assessment Report (JAMAR; [28,29]) and the Patient-Reported Outcomes Measurement Information System (PROMIS; [30,31]). JAMAR is a disease-specific instrument for patients with JIA, whereas PROMIS comprises standardised measures of physical, mental, and social health across paediatric and adult populations.

For the performed analysis, the following JAMAR domains were included: (1) Physical functioning: a total score was calculated from 15 items rated on a 4-point Likert scale (0 = without difficulty to 3 = unable to do; ‘not applicable’ option available) referring to the previous month. Total scores ranged from 0 to 45, with higher scores indicating greater functional impairment. (2) Physical HRQOL and (3) psychosocial HRQOL: each HRQOL domain comprised 5 items rated on a 4-point Likert scale (0 = never to 3 = all the time; ‘not assessable’ option available) referring to the previous month. Subscale scores ranged from 0 to 15, with higher scores indicating poorer HRQOL. (4) Overall well-being, (5) disease activity, and (6) pain intensity were each assessed using a 21-point numeric VAS ranging from 0 to 10, with higher values indicating poorer well-being, higher disease activity, and greater pain intensity, respectively.

For PROMIS, paediatric short-form measures of (1) fatigue (10 items; [32,33]), (2) depressive symptoms (8 items; [34]), (3) anxiety (8 items; [34]), and (4) pain interference (8 items; [35]) were administered. Items were rated on 5-point Likert scales reflecting frequency or intensity, depending on the domain, and referred to the past 7 days. Raw scores were summed and converted into standardised T-scores using PROMIS scoring manuals. T-scores have a mean of 50 and a SD of 10 in the reference population, with higher scores indicating greater levels of the respective construct (eg, higher levels of anxiety).

#### Parent-specific measures

Parents additionally reported their relationship to the child/adolescent (mother or father) and caregiver burden. Caregiver burden was assessed with the Care-related Quality of Life instrument 7-dimension descriptive system [36,37]. A brief self-report scale derived from selected items of this instrument was used in this study. Based on theoretical considerations, 3 items were retained to capture core aspects of caregiver burden: problems related to the caregiver’s mental and physical health, and the ability to manage daily activities. Item selection was guided by a conceptual focus on subjective strain directly associated with caregiving, rather than broader aspects of the caregiving experience. The 3 included items were rated on a 3-point Likert scale ranging from 1 to 3, with higher scores indicating higher perceived burden. A composite score was computed as the mean across items. Internal consistency of the resulting scale was good (Cronbach’s *α* = 0.79).

### Analyses

Complementary statistical approaches were used for the different study aims. First, ICCs were calculated to assess agreement for continuous variables. ICCs were estimated using a 2-way random-effects model with absolute agreement and reported with 95% CIs. Values were interpreted according to established guidelines as follows: <0.5 = poor, 0.5 to 0.75 = moderate, 0.75 to 0.9 = good, and >0.9 = excellent [38].

Second, systematic differences between parent and child reports were assessed. Normality of paired differences (parent minus child) was evaluated using Shapiro-Wilk tests and visual inspection. As JAMAR difference scores were not normally distributed, Wilcoxon signed-rank tests were used. For PROMIS outcomes, paired-samples t tests were applied except for pain, for which the Wilcoxon signed-rank test was used, as the assumption of normality was not met. Effect sizes were calculated to complement significance testing. For independent samples t tests, Cohen’s *d* was computed as a measure of standardised mean differences. For nonparametric tests (Wilcoxon tests), effect sizes were calculated as *r*, derived from the standardised test statistic (z) divided by the square root of the sample size (N). According to commonly used conventions, values of *d* ≈ 0.20, 0.50, and 0.80 indicate small, medium, and large effects, respectively, whereas for *r*, values of ≈ 0.10, 0.30, and 0.50 represent small, medium, and large effects [39].

Third, multinomial regression analyses were conducted. Agreement categories were defined by the difference between parent and child scores in physical and psychosocial HRQOL: differences within ±1 point were classified as agreement (the reference category); larger negative differences indicated worse child-reported HRQOL; and larger positive differences indicated worse parent-reported HRQOL. This cut-off was informed by the observed score distribution, which showed clustering around −1, 0, and +1. Model fit was assessed using likelihood ratio tests and Nagelkerke’s pseudo-R². All analyses were conducted using IBM SPSS Statistics 27.0 (IBM Corp.), with a significance level of *P* < .05.

## RESULTS

Recruitment took place between January 2024 and June 2025. Table 1 displays sample characteristics. For the present study, 88 dyads (72 mothers, 81.8%) with complete parent and patient surveys were included. The mean age of the patients was 13.9 years (SD = 2.2; range 10-18), and 63.3% were females. The most common JIA subtypes were oligoarticular persistent (36.4%) and rheumatoid factor-negative polyarticular JIA (22.7%).

**Table 1:** Characteristics of JIA participants

	Total Sample n = 88
Age, y, mean (SD), range	13.9 (2.2), 10.0-18-0
Gender identity, n (%)	
Male	32 (36.4)
Female	56 (63.6)
Diverse	0
JIA subtype, n (%)	
Oligoarticular JIA (persistent)	32 (36.4)
RF-negative polyarticular JIA	20 (22.7)
Enthesitis-related JIA	12 (13.6)
Oligoarticular JIA (extended)	10 (11.4)
Psoriatic juvenile JIA	5 (5.7)
Systemic JIA	4 (4.5)
RF-positive polyarticular JIA	2 (2.3)
Other/nonclassified JIA	3 (3.4)

JIA, juvenile idiopathic arthritis; RF, rheumatoid factor

Table 2 shows ICC for all JAMAR and PROMIS variables, reflecting the degree of parent-child agreement. ICCs ranged from poor to moderate across PROMIS outcomes and were moderate across JAMAR domains. Agreement tended to be somewhat higher for more observable domains, such as physical HRQOL (ICC = 0.65 [0.51-0.76]), compared with the less directly observable psychosocial HRQOL domain (ICC = 0.50 [0.32-0.64]); however, this pattern was not consistent across all outcomes. Pain demonstrated relatively high agreement (JAMAR, pain intensity: ICC = 0.70 [0.57-0.79]; PROMIS, pain interference: ICC =0.73 [0.60-0.82]) compared with other domains. Lower agreement was observed for internal states such as fatigue (ICC = 0.47 [0.29-0.63]) and anxiety (ICC = 0.47 [0.28-0.62]). Confidence intervals indicated substantial uncertainty, often spanning adjacent interpretative categories.

**Table 2:** Intraclass correlation coefficients of outcome measures

	ICC [95% CI]
JAMAR	
Physical functioning (n = 87)^a^	0.62 [0.48-0.74]
Physical HRQOL (n = 86)^a^	0.65 [0.51-0.76]
Psychosocial HRQOL (n = 88)	0.50 [0.32-0.64]
Overall well-being (VAS) (n = 87)^a^	0.66 [0.52-0.76]
Disease activity (VAS) (n = 88)	0.58 [0.43-0.70]
Pain intensity (VAS) (n = 88)	0.70 [0.57-0.79]
PROMIS	
Fatigue (n = 81)^a^	0.47 [0.29-0.63]
Depression (n = 84)^a^	0.62 [0.47-0.74]
Anxiety (n = 83)^a^	0.47 [0.28-0.62]
Pain interference (n = 80)^a^	0.73 [0.60-0.82]

ICC, intraclass correlation coefficient; HRQOL, health-related quality of life; JAMAR, Juvenile Arthritis Multidimensional Assessment Report; PROMIS, patient-reported outcome information system; VAS, visual analogue scale.

a Missing data due to ‘do not know’ answers or ‘not applicable’; ICC can be interpreted as <0.5 = poor, 0.5 to 0.75 = moderate, 0.75 to 0.9 = good, and >0.9 = excellent [38].

Table 3 presents results from paired-samples t tests examining within-dyad differences between parent and child scores across the considered outcomes; when assumptions were violated, nonparametric alternatives were applied. For most domains, no significant differences emerged. In the JAMAR measures, parent and child reports did not differ significantly in physical functioning, physical and psychosocial HRQOL, overall well-being, or pain intensity (all *P* > .05), and effect sizes were negligible to small (*r* = 0.03-0.12). However, a significant difference emerged for disease activity, with children reporting slightly higher disease activity than parents (*Z* = 2.228, *P* = .026, and *r* = 0.24), indicating a small effect.

**Table 3:** Differences between parent and child reports across JAMAR and PROMIS outcomes

Outcome	Test^a^	Parent: Mean (SD) or Median (Q1-Q3)^b^	Child: Mean (SD) or Median (Q1-Q3)^b^	Statistic	*P*	Effect size Cohens’ *d* or *r*^c^
JAMAR						
Physical Functioning (n = 87)^d^^,^^e^	Wilcoxon	0 (0-3)	0 (0-4)	Z = –0.358	.720	*r* = 0.04
Physical HRQOL (n = 86)^d^^,^^e^	Wilcoxon	1 (0-4)	2 (1-4)	Z = 0.379	.705	*r* = 0.04
Psychosocial HRQOL (n = 88)^e^	Wilcoxon	1 (0-4)	2 (0-4)	Z = 0.246	.806	*r* = 0.03
Overall well-being (n = 87)^d^^,^^e^	Wilcoxon	1 (0-4)	2 (1-4)	Z = 1.047	.295	*r* = 0.11
Disease activity (n = 88)^e^	Wilcoxon	1 (0-3.0)	1 (0-3.9)	Z = 2.228	.026*	*r* = 0.24
Pain intensity (n = 88)^2^	Wilcoxon	1 (0-3.5)	1 (0-3.9)	Z = 1.149	.251	*r* = 0.12
PROMIS						
Fatigue (n = 81)^d^^,^^e^	t test	46.68 (9.92)	44.94 (12.62)	t(80) = 1.347	.182	d = 0.15
Depression (84)^d^^,^^e^	t test	46.30 (9.96)	46.68 (11.31)	t(83) = –0.377	.707	d = -0.04
Anxiety (83)^d^^,^^e^	t test	45.06 (9.84)	43.79 (10.01)	t(82) = 1.126	.263	d = 0.12
Pain interference (80)^d^^,^^e^	Wilcoxon	44.00 (38.00-55.00)	41.65 (34.00-51.70)	Z = –2.596	.009**	*r* = 0.29

HRQOL, health-related quality of life; JAMAR, Juvenile Arthritis Multidimensional Assessment Report; PROMIS, patient-reported outcomes measurement information system.

a JAMAR outcomes were analysed using Wilcoxon signed-rank tests due to non-normality of the difference scores. PROMIS outcomes were analysed using paired-samples t tests or Wilcoxon signed-rank tests, as appropriate based on distributional assumptions.

b Values are presented as mean (SD) for normally distributed data and median (Q1-Q3) for non-normally distributed data.

c Effect sizes are reported as Cohen’s d for t tests and *r* for Wilcoxon signed-rank tests.

d Missing data due to ‘do not know’ answers or ‘not applicable’.

e Higher scores indicate worse outcomes (eg, lower physical functioning, worse HRQOL, more fatigue, etc.).

For the PROMIS outcomes, no significant parent-child differences were found for fatigue, depression, or anxiety (all *P* > .05), with small effect sizes (*d* = −0.04 to 0.15). In contrast, pain interference differed significantly between parent and child reports (*Z* = −2.596, *P* = .009, and *r* = 0.29), suggesting that parents perceived greater pain-related interference than children. Overall, these findings indicate substantial agreement between parent and child assessments, with disagreement primarily limited to disease activity and pain interference.

To examine factors associated with the direction of (dis)agreement between parent- and child-reported HRQOL, 2 multinomial logistic regression models (physical and psychosocial HRQOL) were estimated (Table 4). Both models were statistically significant based on likelihood ratio tests (physical HRQOL: χ²(10) = 28.09, *P* = .002; psychosocial HRQOL: χ²(10) = 25.84, *P* = .004), indicating that the predictors jointly contributed to explaining the outcome. Model fit indices suggested an acceptable fit for both models (physical HRQOL: Nagelkerke’s R² = .33; psychosocial HRQOL: Nagelkerke’s R² = .30). Higher self-reported depressive symptoms in children were associated with an increased likelihood of children reporting worse HRQOL than their parents in both HRQOL domains (psychosocial: odds ratio [OR] = 1.09, *P* = .003; physical: OR = 1.09, *P* = .004). In contrast, higher parent-reported caregiver burden was associated with a greater likelihood that parents would report worse HRQOL than their children in both psychosocial (OR = 1.53; *P* = .031) and physical (OR = 1.88; *P* = .004) HRQOL. Disease activity and age were not significantly associated with the direction of disagreement in either model. Gender (male vs female) was significantly associated with the direction of disagreement only for psychosocial HRQOL, with male patients showing higher odds of reporting worse HRQOL than their parents compared with female patients (OR = 3.65; *P* = .042).

**Table 4:** Predictors of parent-child disagreement direction in physical and psychosocial HRQOL: multinomial logistic regression analyses

	Physical HRQOL				Psychosocial HRQOL			
	Child-worse vs Agreement		Parent-worse vs Agreement		Child-worse vs Agreement		Parent-worse vs Agreement	
Predictors	OR (95% CI)	*P*	OR (95% CI)	*P*	OR (95% CI)	*P*	OR (95% CI)	*P*
Age	0.93 (0.70-1.24)	.616	0.90 (0.68-1.19)	.459	1.15 (0.87-1.51)	.336	0.90 (0.70-1.18)	.451
Male (ref: female)	1.26 (0.34-4.66)	.731	1.16 (0.33-4.08)	.818	**3.65 (1.05-12.77)**	**.042**	2.03 (0.63-6.49)	.233
Disease Activity (clinician rating)	1.05 (0.78-1.42)	.750	1.22 (0.93-1.61)	.151	0.99 (0.75-1.31)	.958	1.03 (0.78-1.35)	.839
Child depression (PROMIS; self-rating JIA-patient)	**1.09 (1.03-1.16)**	**.004**	0.99 (0.93-1.06)	.852	**1.09 (1.03-1.16)**	**.003**	1.01 (0.95-1.07)	.759
Caregiver burden	1.43 (0.88-2.30)	.147	**1.88 (1.22-2.90)**	**.004**	0.79 (0.47-1.35)	.392	**1.53 (1.04-2.25)**	**.031**

HRQOL, health-related quality of life; JIA, juvenile idiopathic arthritis; OR, odds ratio; PROMIS, patient-reported outcomes measurement information system.

## DISCUSSION

The present study examined the level of agreement and systematic differences at the group level between P-PROMs and PROMs across multiple domains, as well as predictors of disagreement in children with JIA. The hypotheses were partly supported. First, agreement was generally moderate, with somewhat higher ICCs for more observable domains (eg, physical HRQOL) than for more subjective domains (eg, psychosocial HRQOL, fatigue, and anxiety). However, contrary to expectations, agreement for pain intensity and pain interference was relatively high, despite pain typically being considered a subjective experience. Second, consistent with our hypotheses, no systematic pattern of parent-child differences emerged across domains. For most domains, no significant differences were observed, whereas in 2 domains (disease activity and pain interference) parent-child differences varied in direction. Finally, child depressive symptoms were associated with a higher likelihood of children rating their HRQOL worse than their parents, whereas caregiver burden was associated with a higher likelihood of parents rating the child’s HRQOL worse than the children themselves.

Our ICC analyses indicated moderate to good agreement for pain-related indicators. Similarly, some earlier studies have reported moderate to good agreement for pain [[14], [15], [16]] and higher agreement for this construct than for some more observable dimensions (eg, fine motor functioning [16] or daily activities [15]). This comparatively high agreement (although still moderate) may be explained by the central role of pain in JIA. Accordingly, it has been suggested that domains more central to a given health condition may promote ongoing parent-child communication and, consequently, a shared understanding [3]. Furthermore, it should be considered that pain in JIA is closely linked to observable functional limitations, such as limping and morning stiffness, which may contribute to higher levels of agreement compared with other domains. However, findings across studies are mixed, and some earlier work found low agreement in pain assessment in this patient population [19,21].

Despite moderate to good agreement for pain, the overall moderate agreement across domains at the individual level (including physical functioning, physical and psychosocial HRQOL, overall well-being, depression, and pain interference) suggests that parent and child reports are not interchangeable but rather provide complementary perspectives on paediatric outcomes. Therefore, both informants should be considered jointly, particularly given that parents’ perceptions often guide healthcare utilisation and may lead to unmet needs if children’s difficulties are underestimated [2]. Considering both child and parent perspectives is also consistent with the principles of the United Nations Convention on the Rights of the Child, particularly the child’s right to be heard in matters affecting them and the requirement to consider their best interests [40].

From a clinical perspective, relying solely on parental reports may contribute to suboptimal treatment decisions [12], especially for complex domains such as fatigue or anxiety. Clinicians should therefore assess both perspectives independently and pay particular attention to disagreement, as it may signal increased burden of child or parent and help identify families who may benefit from targeted behavioural or family-based interventions [12]. Discrepant ratings may provide a starting point for discussions about the child’s symptoms, daily functioning, emotional well-being, and illness-related experiences. Exploring such discrepancies may help clinicians identify unmet needs, differences in illness perceptions, communication difficulties, or psychosocial burdens within the family that might otherwise remain unnoticed. For example, when substantial discrepancies emerge in psychosocial domains, clinicians may consider systematically exploring potential depressive symptoms in the child or elevated caregiver burden in the parent and, where appropriate, refer to psychosocial services.

These clinical implications align with our findings that higher levels of depressive symptoms in children were associated with more negative self- versus proxy-reported physical and psychosocial HRQOL, which is consistent with previous JIA studies [[10], [11], [12]]. Several explanations may account for this association. Depressive symptoms may be associated with genuinely poorer physical and psychosocial functioning, but they may also influence how children perceive, recall, and report their experiences. Specifically, children’s negative emotional state may systematically amplify the perceived severity of health problems [12]. Moreover, our study found an association between caregiver burden and a higher likelihood that parents report worse HRQOL than the child, which aligns with earlier research [2]. Caregiver burden may reflect greater caregiving demands due to the child’s disease and treatment requirements. However, it may also reflect psychosocial strain or be associated with depressive symptoms in caregivers, which could influence parental perceptions and result in more negative proxy reports. From a clinical perspective, if caregivers report a high burden and a lower proxy-reported HRQOL than the child reports themselves, this discrepancy may indicate that the caregiver’s own well-being and support needs also warrant attention. Together, these findings point towards informant-specific influences, whereby children’s internal emotional states and parents’ caregiving experiences may shape their respective perceptions. However, given the cross-sectional design of this study, the direction of these associations remains unclear. At the same time, discrepancies between child and parent reports may not only be explained by child- and parent-specific characteristics. Family-level factors may also influence parent-child agreement. Previous research suggests that discrepancies between child and parent reports may partly reflect differences in access to information and impaired communication processes within families rather than inaccurate reporting by either informant [2,3,6]. Parents may be more aware of experiences that are openly discussed with their children, whereas less observable symptoms such as emotional distress, fatigue, or worries may remain unnoticed when communication is limited [2,6].

Regarding gender identity, our study found that male participants were more likely than female participants to report poorer psychosocial HRQOL than their parents. As psychosocial difficulties are less observable, parents may have fewer cues to detect them. This may be particularly pronounced in male adolescents, who tend to self-disclose less [41], potentially limiting parental awareness of psychosocial distress captured by the JAMAR HRQOL assessment. However, this interpretation should be treated with caution, as previous studies have reported inconsistent findings. For example, Lundberg et al. [22] observed more pronounced differences between girls and their parents, with parents reporting lower physical and psychosocial HRQOL, whereas differences were largely absent for boys. Moreover, other studies did not identify any effect of sex on disagreement [9,21]. These inconsistencies may partly reflect methodological differences, including the measurement instruments used, sample characteristics, and analytical approaches.

Several limitations regarding the current study should be considered. First, parent-child agreement may have been overestimated, particularly among younger children. In cases where children did not have their own email address, survey links were distributed via parents, who may have been present during completion or assisted their children, thus potentially influencing responses. Second, the PROMIS measures included a ‘don’t know’ response option. Cases in which parents selected this option were excluded from the analyses. In these instances, agreement may have been lower compared with dyads providing definitive responses, potentially leading to an underestimation of disagreement between parent and child reports. At the same time, selecting ‘don’t know’ may itself reflect limited parental awareness of the child’s internal experiences and thus represent a meaningful form of parent-child disagreement. However, this likely had only a minor impact, as this response option was selected by only a few participants. Third, observed parent-child disagreement may not solely reflect genuine differences in perceptions. Some discrepancies may also be attributable to methodological factors inherent to self- and proxy-reported measures, including differences in item interpretation, response tendencies, recall processes, or contextual influences. In addition, potential psychometric differences between child and proxy versions, such as differential item functioning, cannot be ruled out. Given the relatively small sample size, formal measurement invariance or differential item functioning analyses were beyond the scope of this study. Fourth, the use of informant-specific predictors (ie, child-reported depressive symptoms and parent-reported caregiver burden) in models predicting disagreement scores introduces a degree of structural overlap, as predictors are inherently linked to 1 component of the outcome. As a result, associations may partly reflect shared method variance or informant-specific bias rather than independent effects, and findings should be interpreted with caution. Fifth, the relatively small analytical sample required limiting the number of predictors included in the regression models, which may have reduced the ability to detect smaller effects. Finally, the relatively good overall health status and low symptom burden in our sample may have led to restricted variability in several outcomes, potentially attenuating observed associations. This should be considered when interpreting the magnitude of effects. Moreover, the generalisability of our findings may be limited, as the sample may underrepresent children with more severe disease or those recently diagnosed. These groups may exhibit higher symptom levels and potentially different patterns of parent-child (dis)agreement.

## CONCLUSIONS AND RECOMMENDATIONS FOR FUTURE RESEARCH

In conclusion, the present study highlights that parent-child agreement in HRQOL among children with JIA is only moderate and varies across domains, indicating that parent and child reports capture related but distinct aspects of the child’s experience. While no consistent group-level differences between informants emerged, disagreement in HRQOL was associated with child depressive symptoms and caregiver burden, suggesting that both child and parent characteristics shape perceptions of this outcome. These findings underscore the importance of incorporating both child self-reports and parent proxy reports into clinical assessment and research, particularly for less observable psychosocial domains. Rather than being treated as measurement error, parent-child disagreement may provide clinically meaningful information about family functioning and emotional burden, thereby helping to identify families who may benefit from additional psychosocial support. Clinically, a structured review of child-parent discrepancies during consultations may support shared decision-making, ensure that the child’s voice is heard, and guide tailored referrals to psychosocial interventions where needed.

Future research should investigate the clinical utility of parent-child disagreement, including whether combining child and parent reports or considering disagreement itself improves the prediction of clinically relevant outcomes and supports patient- and family-centred care. Longitudinal and multi-informant studies are needed to clarify the role of family-level factors, such as communication, relationship quality, family functioning, and parental involvement, in shaping agreement and disagreement over time. Finally, larger studies should examine the measurement equivalence of self- and proxy-reported measures to better distinguish true perceptual differences from methodological sources of disagreement.

## CRediT authorship contribution statement

**Michelle Dey:** Writing – review & editing, Writing – original draft, Software, Project administration, Methodology, Investigation, Formal analysis, Data curation, Conceptualization. **Lisa Pedrotti:** Writing – review & editing, Project administration, Investigation. **Federica Vanoni:** Writing – review & editing, Project administration, Investigation. **Tatjana Welzel:** Writing – review & editing, Project administration, Investigation. **Julia Dratva:** Writing – review & editing, Supervision, Software, Project administration, Methodology, Investigation, Conceptualization. **Rotraud K. Saurenmann:** Writing – review & editing, Supervision, Resources, Project administration, Methodology, Investigation, Funding acquisition, Conceptualization.

## Competing interests

All authors declare they have no competing interests.

## Appendix Supplementary materials

Image, application 1
