# Supplementary material for: Parent-child (dis)agreement in patient-reported outcomes in juvenile idiopathic arthritis: the role of child depression and caregiver burden
Source: EULAR Rheumatol Open. 2026 Sep 22;2(4):100253. doi: 10.1016/j.ero.2026.100253 (PMC13694729; doi:10.1016/j.ero.2026.100253)
Supplement: Supplementary file 1 [file mmc1.docx]

Supplementary Material: Participating centers in the Swiss-PAVE study

| Centre | Local Project Leader |
| --- | --- |
| Pediatric Rheumatology, University Children’s Hospital Basel | Andreas Wörner |
| Pediatric Rheumatology, University Children’s Hospital Bern | Klaus Tenbrock |
| Pediatric Rheumatology, University Children’s Hospital Zurich | Elvira Cannizzaro |
| Center for Pediatrics and Adolescent Medicine, Cantonal Hospital Winterthur | Traudel Saurenmann |
| Pediatric Rheumatology, Department of Pediatrics, Cantonal Hospital Lucerne | Daniela Kaiser |
| Pediatric Rheumatology, Cantonal Hospital Aarau | Florence Aeschlimann |
| Pediatric Rheumatology, Eastern Switzerland Children’s Hospital | Christian Huemer |
| Pediatric Rheumatology, Cantonal Hospital of Grisons | Eva Adank |
| Pediatric Rheumatology, Ente Ospedaliero Cantonale (EOC) | Federica Vano |
